# Supplementary material for: Manipulating electron redistribution to achieve electronic pyroelectricity in molecular [FeCo] crystals
Source: Nat Commun. 2021 Aug 10;12:4836. doi: 10.1038/s41467-021-25041-4 (PMC8355315; doi:10.1038/s41467-021-25041-4)
Supplement: Supplementary file 1 — Supplementary Information [file 41467_2021_25041_MOESM1_ESM.pdf]

Supplementary Information

Manipulating electron redistribution to achieve electronic pyroelectricity in molecular [FeCo] crystals

Pritam Sadhukhan[1,#], Shu-Qi Wu[1,#], Jeremy Ian Long[1], Takumi Nakanishi[1], Shinji Kanegawa[1,*], Kaige Gao[2], Kaoru Yamamoto[3], Hajime Okajima[4], Akira Sakamoto[4], Michael L. Baker[5,6], Thomas Kroll[7], Dimosthenis Sokaras[7], Atsushi Okazawa[8], Norimichi Kojima[9], Yoshihito Shiota[1], Kazunari Yoshizawa[1], and Osamu Sato[1,*]

[1]Institute for Materials Chemistry and Engineering & IRCCS, Kyushu University, 744 Motooka, Nishi-ku, Fukuoka 819-0395, Japan.

[2]College of Physical Science and Technology, Yangzhou University, Jiangsu 225009, P. R. China.

[3]Department of Applied Physics, Okayama University of Science, Okayama 700-0005, Japan

[4]Graduate School of Science and Engineering, Aoyama Gakuin University, 5-10-1 Fuchinobe, Chuo-ku, Sagamihara, Kanagawa 252-5258, Japan.

[5]The Department of Chemistry, The University of Manchester, Manchester M13 9PL, UK.

[6]The Department of Chemistry, The University of Manchester at Harwell, Didcot OX11 0FA, UK.

[7]Stanford Synchrotron Radiation Lightsource, SLAC National Accelerator Laboratory, Stanford University, Menlo Park, California 94025, United States.

[8]Division of Chemistry, Institution of Liberal Education, Nihon University School of Medicine, 30-1 Oyaguchi Kamimachi, Itabashi-ku, Tokyo 173-8610, Japan.

[9]Department of Basic Science, Graduation School of Arts and Sciences, The University of Tokyo, 3-8-1 Komaba, Meguro-ku, Tokyo 153-8902, Japan.

[#]P.S. and S.-Q.W. contributed equally.

Correspondence and requests for materials should be addressed to S. K. (email: kanegawa@cm.kyushu-u.ac.jp) or to O. S. (email: sato@cm.kyushu-u.ac.jp).

Materials and Methods

All solvents and reagents were used as received from Sigma-Aldrich company.

Synthesis of enantiopure [Zn(AcO)(*RR*-cth)](PF_6): A mixture of Zn(AcO)$_2$ (550 mg, 3.0 mmol) and *RR*-cth (850 mg, 3.0 mmol) in EtOH (10 mL) was heated to 60°C under a N_2 atmosphere. After stirring at this temperature, solid NH_4PF_6 (540 mg, 3.3 mmol) was added to the solution. [Zn(AcO)(*RR*-cth)](PF_6) then gradually precipitated within an hour. After the reaction mixture was cooled in an ice-bath, the precipitate was collected by filtration and washed with cold EtOH followed by Et_2O.

Synthesis of [(Co(*SS*-cth))(Zn(*RR*-cth))(μ-dhbq)](PF_6)$_2$: This compound was synthesized by sequential addition of equimolar [Co(AcO)(*SS*-cth)](PF_6) and [Zn(AcO)(*RR*-cth)](PF_6) in the basic solution of 3,5-dihydroxy-1,4-benzoquinone in the same manner as described for the synthesis of [(Fe(*RR*-cth))(Co(*SS*-cth))(μ-dhbq)](PF_6)$_2$.

Synthesis of [(Co(*SS*-cth))(Zn(*RR*-cth))(μ-dhbq)](PF_6)$_3$: To a solution of [(Co(*SS*-cth))(Zn(*RR*-cth))(μ-dhbq)](PF_6)$_2$ (0.3 mmol) dissolved in MeCN (60 mL), solid $AgPF_6$ (76 mg, 0.3 mmol) and H_2O (1 mL) were added. After stirring for 10 min, the mixture was filtered to remove Ag. The filtrate was evaporated to dryness under reduced pressure and the crude product was collected as a solid and washed with a small amount of H_2O. The crude product was recrystallized from a mixed solvent of several drops of MeCN and hot H_2O/MeOH to afford crystals suitable for pyroelectric measurements. Elemental analysis calcd. for $C_{38}H_{74}N_8O_4F_{18}P_3ZnCo$: C: 36.04, H: 5.89, N: 8.85; found: C: 36.04, H: 5.95, N: 8.86. ESI MS (Supplementary Fig. 14): *m/z* = 1121.3 $\{[ZnCo](PF_6)_2\}^+$ (calcd for $C_{38}H_{74}N_8O_4ZnCoP_2F_{12}$: 1121.11).

X-ray diffraction measurements and crystal structural determination

Diffraction data were collected at 60 K under a helium gas stream and at 100, 150, 200, 250, and 300 K under a cold nitrogen gas stream on a Rigaku FR-E+ diffractometer equipped with a HyPix-6000 area detector and multi-layer mirror monochromated Mo-$K\alpha$ radiation (λ = 0.71073 Å). The

structures were solved by a direct method and refined *via* full-matrix least-squares on F^2 using the SHELX program[1] implemented in the OLEX2 program[2] with anisotropic thermal parameters for all non-hydrogen atoms. The hydrogen atoms were geometrically added and refined by the riding model.

Magnetic measurements

Magnetic susceptibility measurements were performed on a quantum design SQUID magnetometer (MPMS-5S). Polycrystalline samples (ca. ~10 mg) were loaded into a gelatin capsule, which was fixed on a sample rod with a plastic straw. Measurements were conducted with H = 1000 Oe in the temperature range of 5–300 K.

Mössbauer spectroscopy

The ^{57}Fe Mössbauer spectra were recorded on a constant acceleration spectrometer with a ^{57}Co/Rh source in the transmission mode. The measurements were performed in a closed-cycle helium refrigerator (Iwatani Industrial Gases Corp.) and a conventional Mössbauer spectrometer (Topologic Systems). All isomer shifts are reported relative to α-Fe at room temperature. The Mössbauer spectra were fitted with the least-squares fitting program MossWinn 4.0.

IR spectroscopy

Temperature-dependent IR spectra were recorded by using an FT-IR spectrophotometer (VERTEX 70, Bruker) equipped with a closed-cycle helium refrigerator cryostat (Nagase Techno-Engineering). The ground-powdered samples were held between grained and flat CaF_2 plates.

Density functional theory (DFT) calculations

DFT calculations for the [FeCo] dinuclear complex in the singlet, triplet, quintet and septet spin states were performed by unrestricted DFT (UDFT) implemented in the Gaussian 09 program package[3]. To remove structural inaccuracies from incomplete spin transitions mentioned above, the DFT-optimized structures were used to compare the relative energies of different spin states and their corresponding vibrational spectra. For the Fe and Co atoms, the (14s9p5d)/[9s5p3d] primitive

set of the Wachters–Hay basis[4,5] with one polarization f-function was used, and for the H, C, N, and O atoms, the D95 basis set[6] was used. Whereas the relative energies of different spin states play a notable role in a multi-spin system, it is well known that DFT methods are less accurate at predicting small energy gaps between different spin states. The B3LYP functional[6] is often used to predict the geometry and spectroscopic properties of systems containing transition metals; however, this tends to overestimate the stability of the high-spin state[7]. Conversely, calculations based on the pure functional tend to overestimate the stability of the low-spin state. We used the B3LYP* hybrid functional for the calculations for the estimation of accurate energy differences between different spin states. This functional is a re-parameterized version of the B3LYP hybrid functional originally suggested by Reiher and coworkers[8]. Vibrational analyzes were performed for all spin states to ensure that no imaginary frequencies existed for all optimized geometries. The calculated IR spectra were scaled by 0.985 for comparison with the experimental results.

Supplementary Note 1 | Nomenclature

Although the Enemark-Feltham nomenclature is conventionally used to describe complexes with strong metal-ligand covalent interactions, here for simplicity, we will use the limiting configurations, [$Fe^{3+}{}_{LS}$–dhbq^{3-}–$Co^{3+}{}_{LS}$], [$Fe^{3+}{}_{HS}$–dhbq^{3-}–$Co^{3+}{}_{LS}$] and [$Fe^{2+}{}_{HS}$–dhbq^{2-}–$Co^{3+}{}_{LS}$], to denote their electronic structures and an extra superscript C (standing for covalent) is used to remind the readers of the presence of strong covalency in such states.

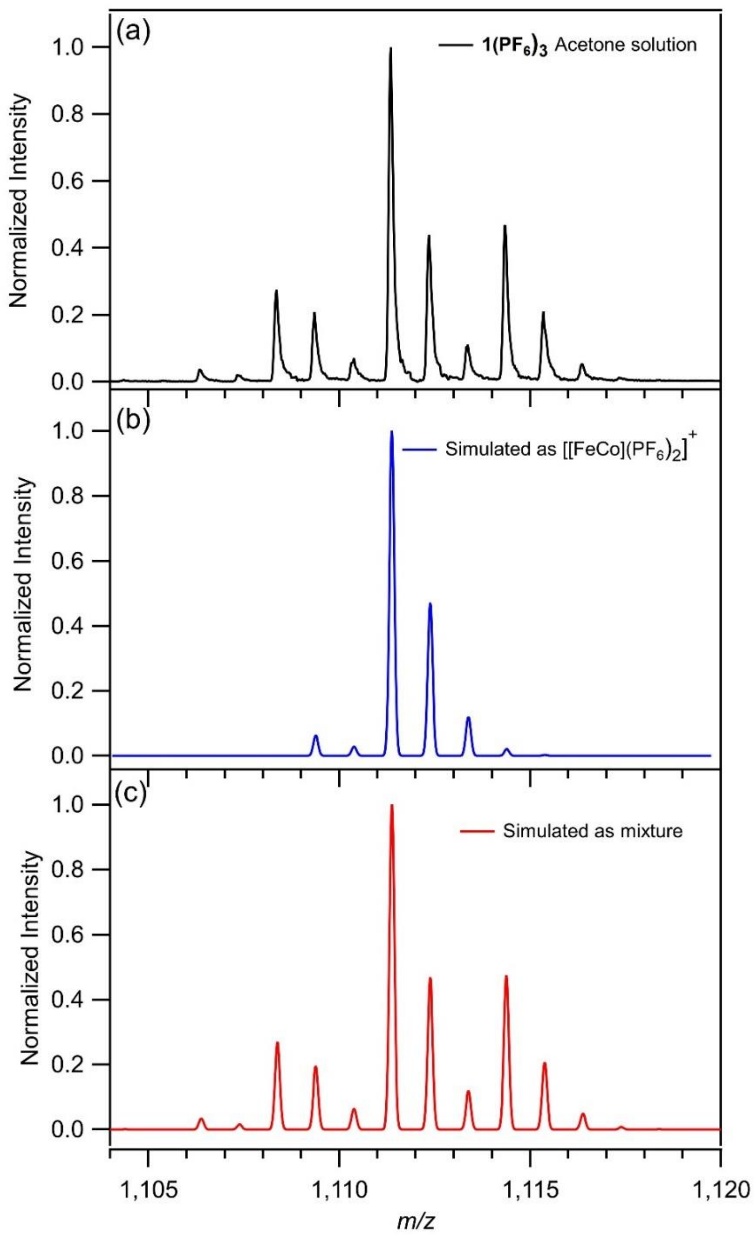

Supplementary Fig. 1 | ESI-MS spectrum of 1(PF_6)$_3$ in acetone. (a) The spectrum exhibits the molecular peak of [**1**(PF_6)$_2$]$^+$ (m/z = 1111.5). (b) Isotopic distribution simulation of [[FeCo](PF_6)$_2$]$^+$($C_{38}H_{74}N_8O_4FeCoP_2F_{12}$). (c) Simulation of the mixture [[FeCo](PF_6)$_2$]$^+$/[[FeFe](PF_6)$_2$]$^+$/[[CoCo](PF_6)$_2$]$^+$ = 1.00/0.27/0.46. It should be noted that, even though [FeFe] and [CoCo] species are generated in solution, the selective crystallization of the [FeCo] species is supported by magnetometry and Co K-edge X-ray absorption spectroscopy. Indeed, there is no sign of valence tautomeric behavior from [CoCo] species in both measurements.

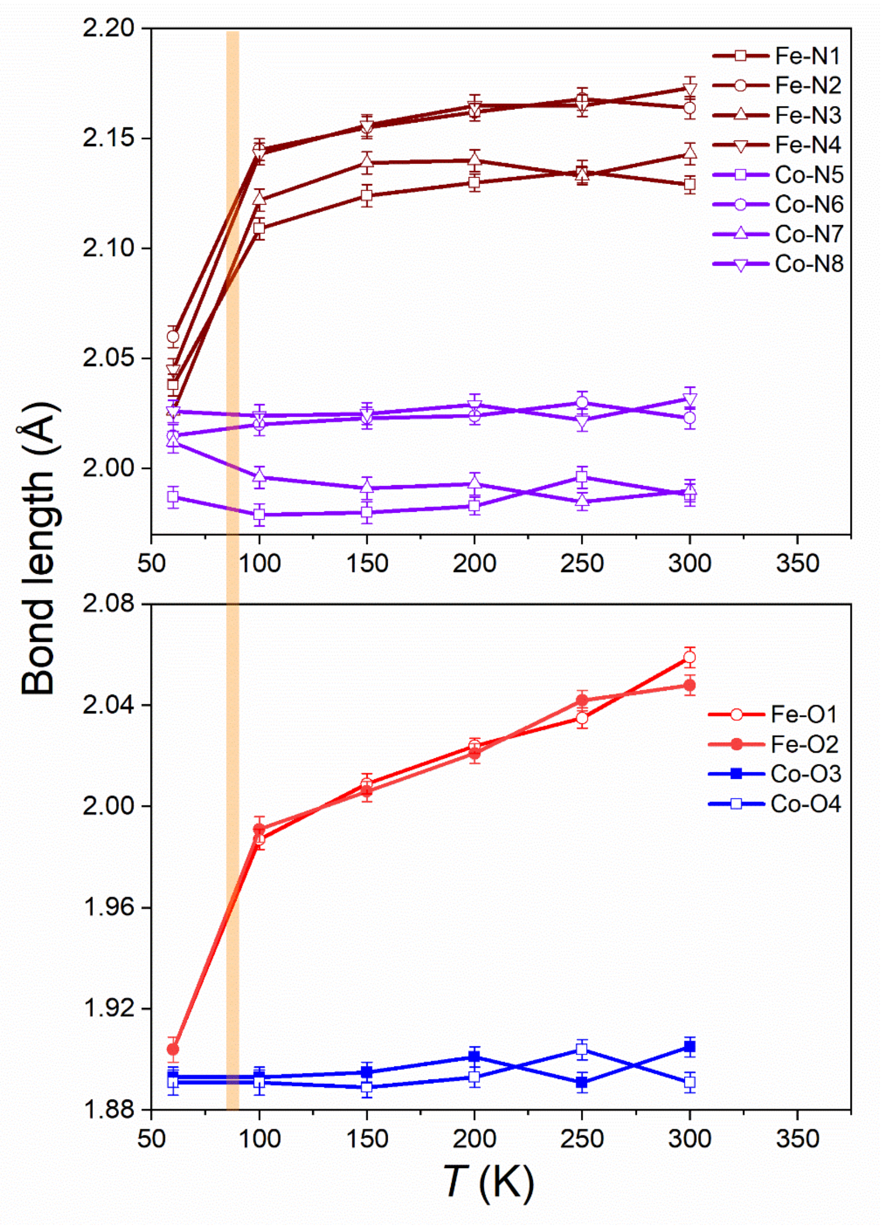

Supplementary Fig. 2 | Changes in metal–ligand bond lengths with temperature for complex 1(PF_6)$_3$. Numbers associated with O and N represent corresponding atoms in CIF.

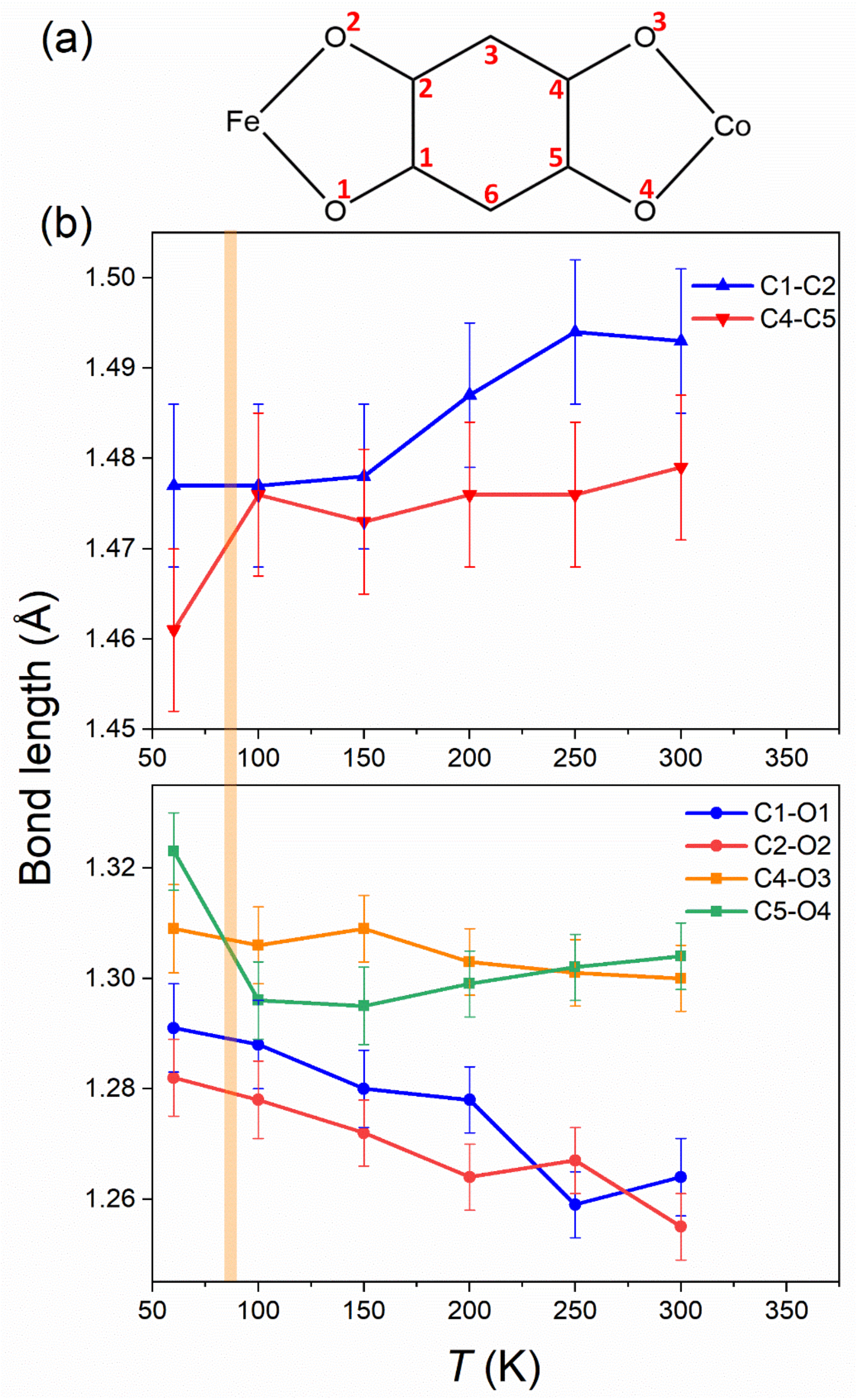

Supplementary Fig. 3 | (a) Schematic indication of the specific bonds for complex 1(PF_6)$_3$. (b) Changes in some specific C–C and C–O bond lengths with temperature for complex 1(PF_6)$_3$.

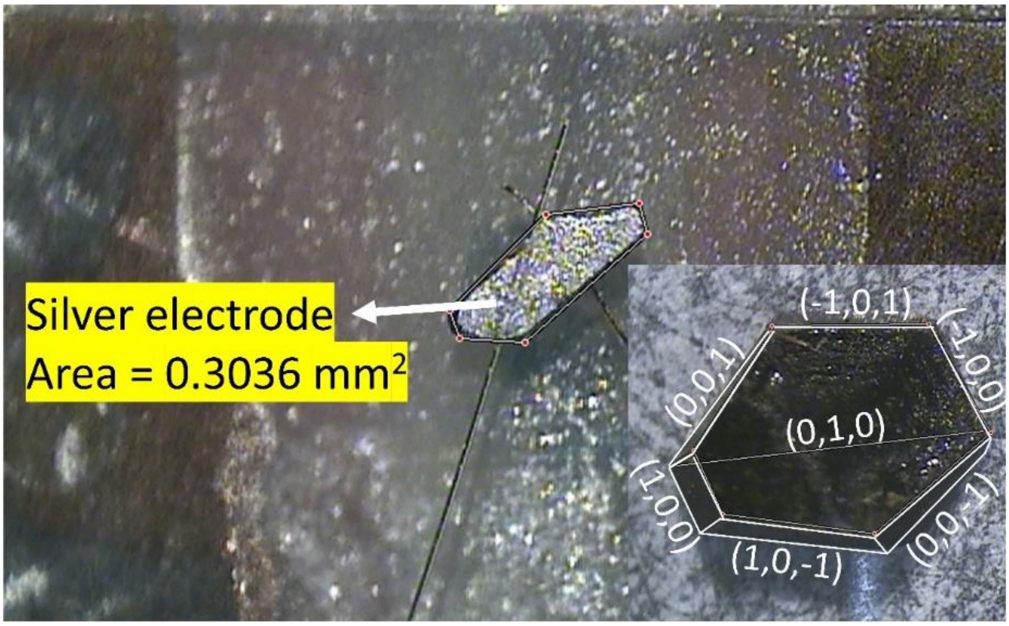

Supplementary Fig. 4 | The single-crystal of $1(PF_6)_3$ used in pyroelectric measurement (inset- crystal phases are marked on a block-shape single crystal).

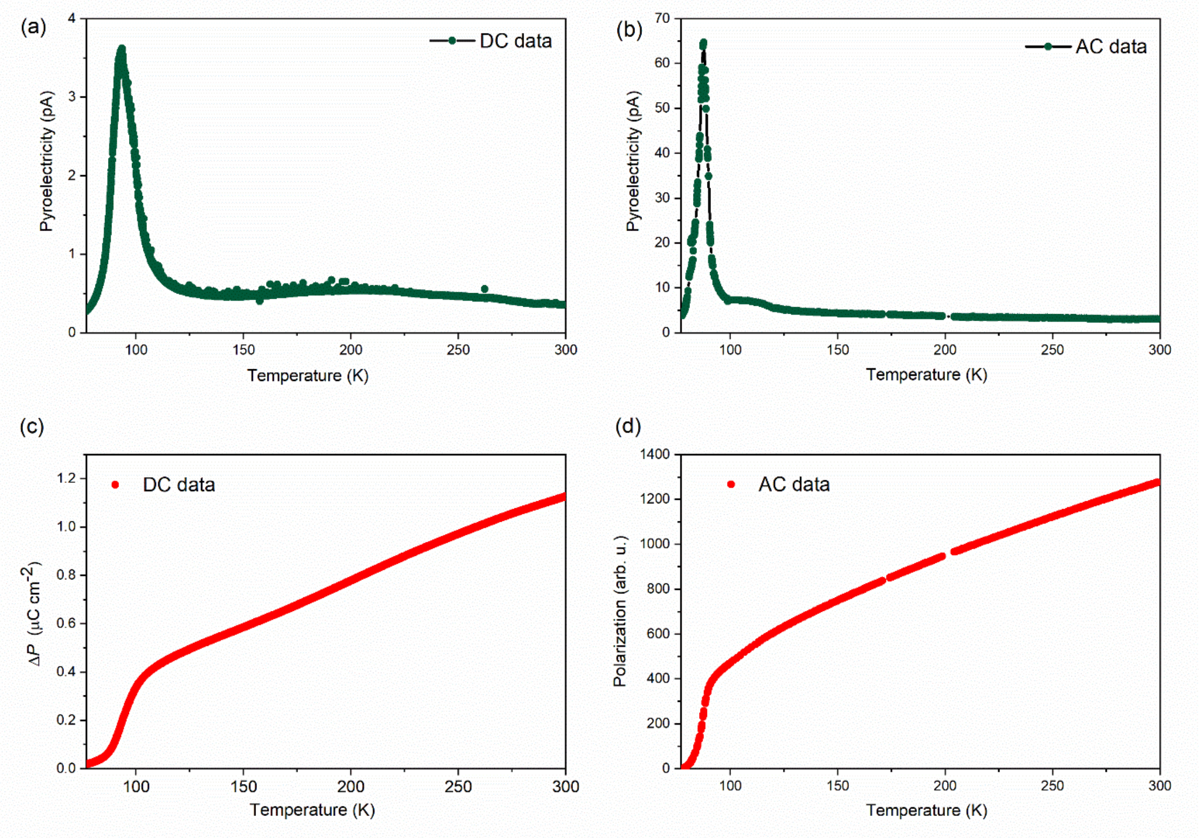

Supplementary Fig. 5 | Qualitative comparison between pyroelectric responses of $1(PF_6)_3$ measured via (a) continuous temperature ramping mode (DC mode) and (b) continuous oscillation mode (AC mode).[9] (c) and (d) show the electric polarization obtained from the respective pyroelectric data. Specific heat of the sample used to estimate the polarization in (d) is considered to be constant with respect to temperature change.

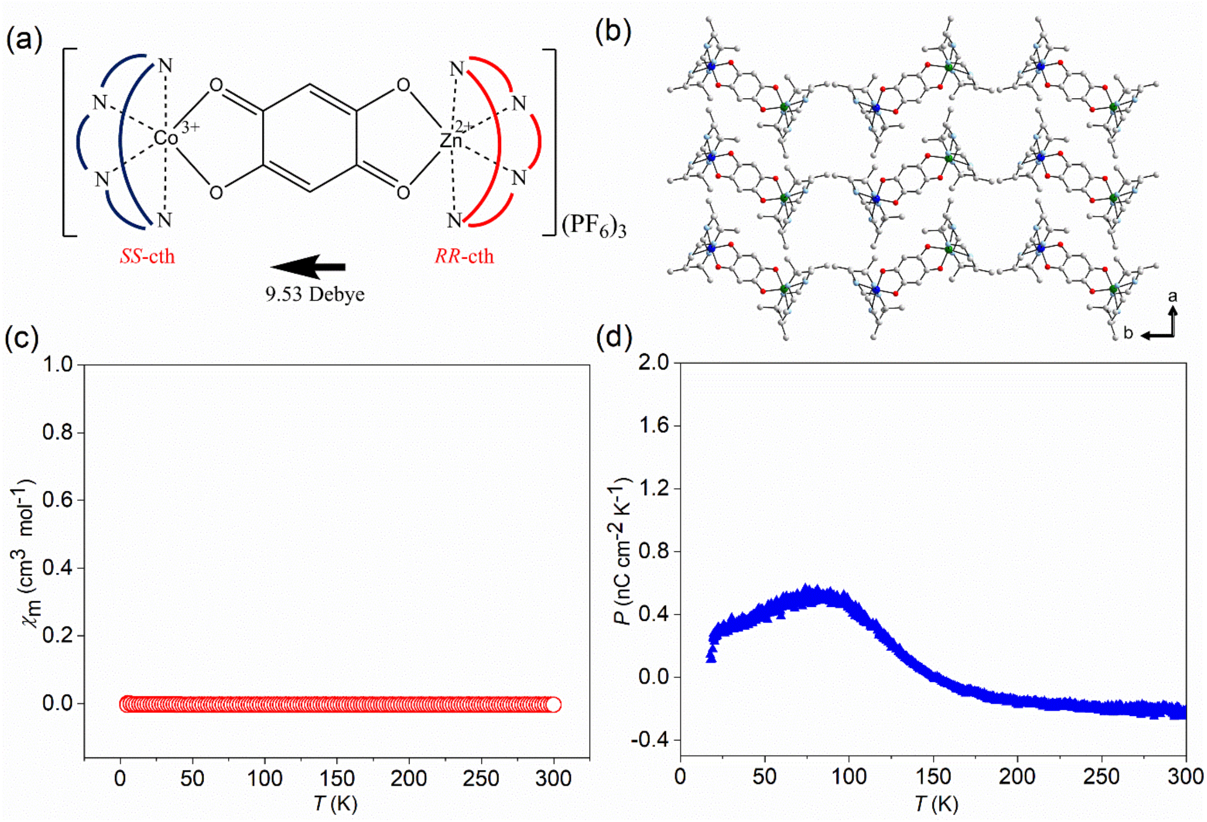

Supplementary Fig. 6 | Characterization of reference compound [ZnCo](PF_6)$_3$]. (a) Molecular structure and calculated dipole moment of [(Zn(*RR*-cth))(Co(*SS*-cth)(μ-dhbq)](PF_6)$_3$: [ZnCo](PF_6)$_3$. (b) Molecular packing of [ZnCo](PF_6)$_3$. [Zn(green), Co(blue), O(red), C(gray)] (c) Temperature dependence of magnetic susceptibilities displayed as a plot of χ_m vs *T*. (d) Temperature dependence of the pyroelectric coefficient for [ZnCo](PF_6)$_3$.

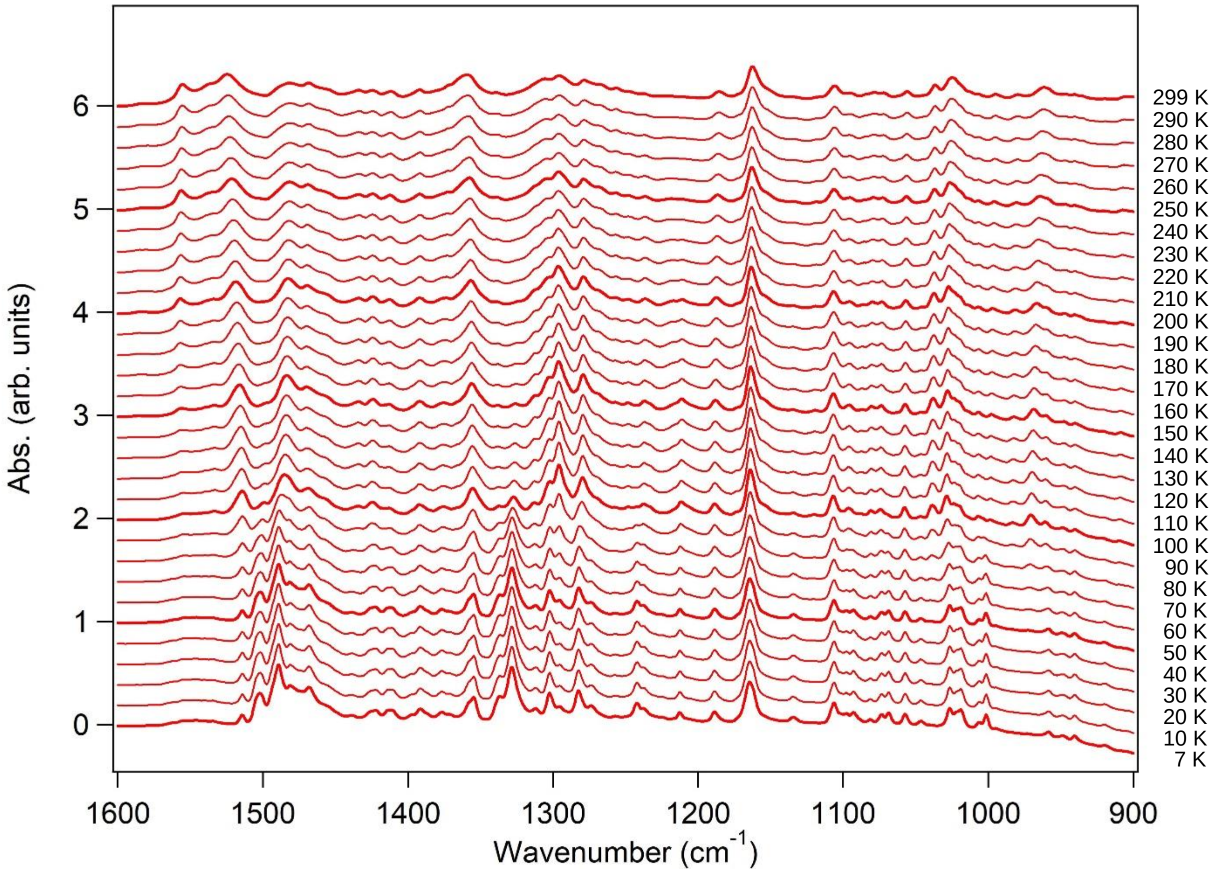

Supplementary Fig. 7 | Variable-temperature infrared absorption spectra in the range of 900–1600 cm^{-1} for a temperature range from 299 to 7 K.

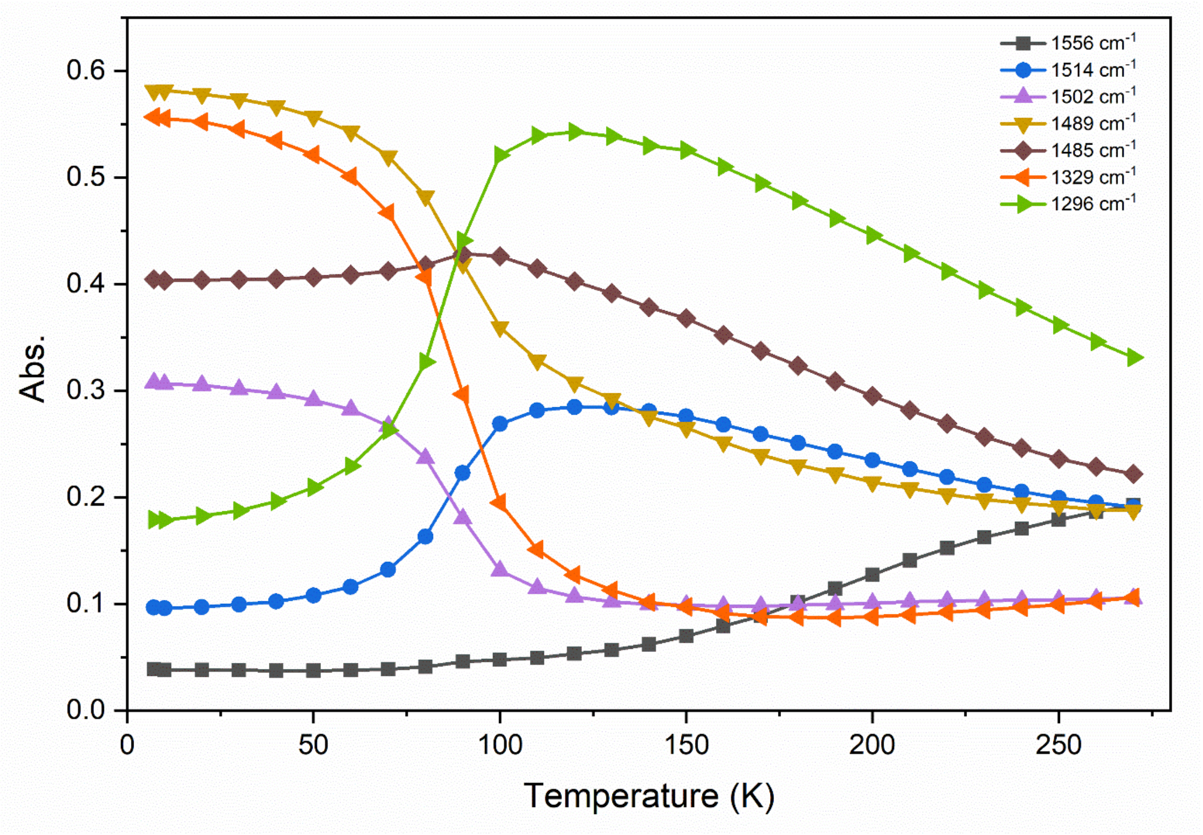

Supplementary Fig. 8 | Variable-temperature infrared absorption change at characteristic wavenumbers for complex 1(PF_6)$_3$.

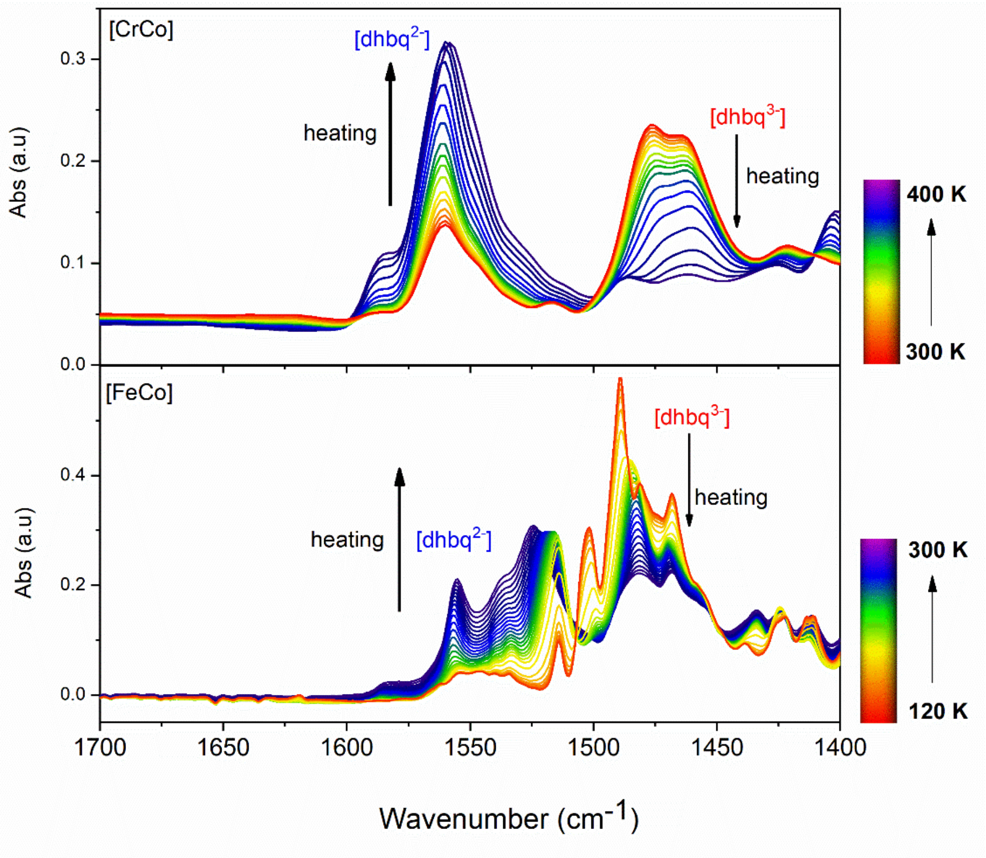

Supplementary Fig. 9 | Comparison of IR absorption spectra between [FeCo] complex and valence tautomeric [CrCo] complex; [CrCo] complex = [(Cr(*SS*-cth))(Co(*RR*-cth))(μ-dhbq)]$(PF_6)_3$. The [CrCo] complex exhibits valence tautomeric transition at 360 K, where low and high temperature phases have the structures of [Cr^{3+}–$dhbq^{3-}$–Co^{3+}_{LS}] and [Cr^{3+}-$dhbq^{2-}$-Co^{2+}_{HS}], respectively.

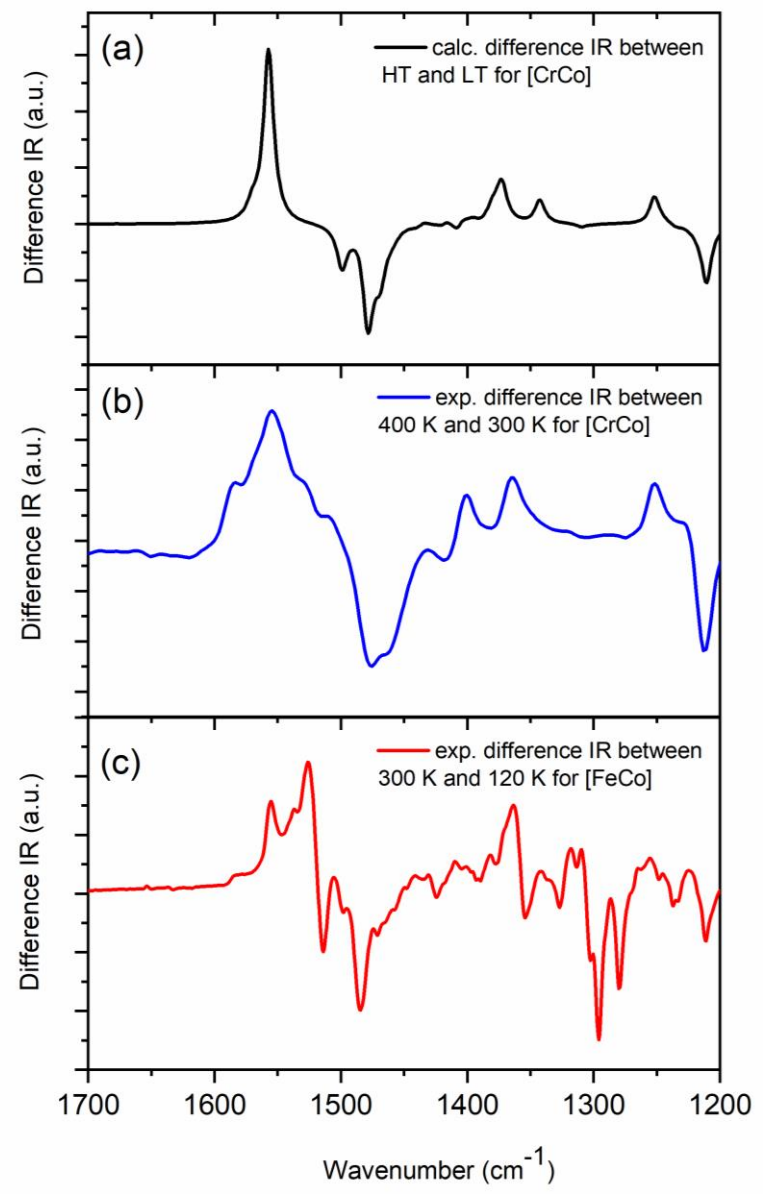

Supplementary Fig. 10 | Comparison of difference IR spectra between [FeCo] system and valence tautomeric [CrCo] system. (a) Difference IR abs spectrum obtained by DFT calculation for [CrCo] VT complex, (b) Experimental data of difference IR abs spectrum between 400 and 300 K for [CrCo] VT complex, (c) Experimental data of difference IR abs spectrum between 300 and 120 K for [FeCo] complex.

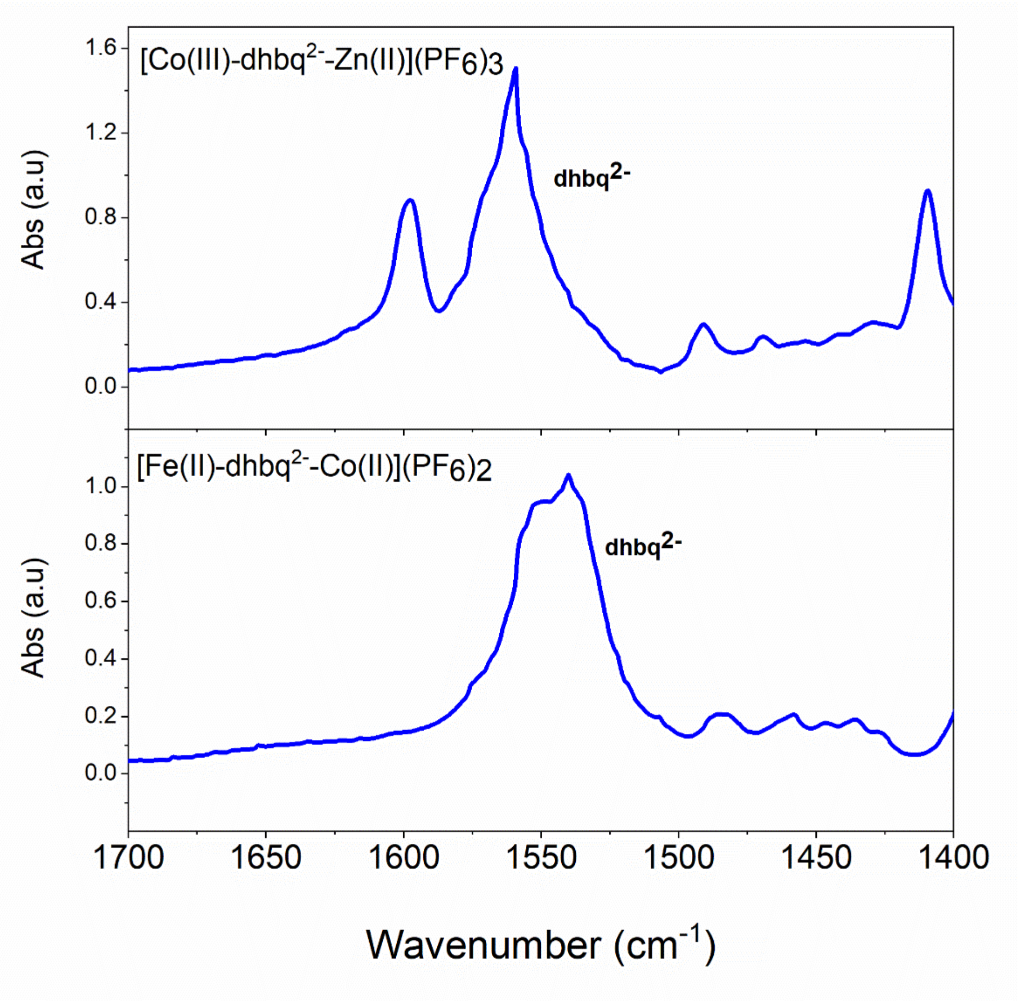

Supplementary Fig 11 | Comparison of IR spectra (recorded at 300 K) between reference complexes $[Co^{III}\text{-dhbq}^{2-}\text{-}Zn^{II}](PF_6)_3$ and $[Fe^{II}\text{-dhbq}^{2-}\text{-}Co^{II}](PF_6)_2$ indicating peak position at around 1540 cm^{-1} characteristic of bridging $[\text{dhbq}^{2-}]$ ligand.

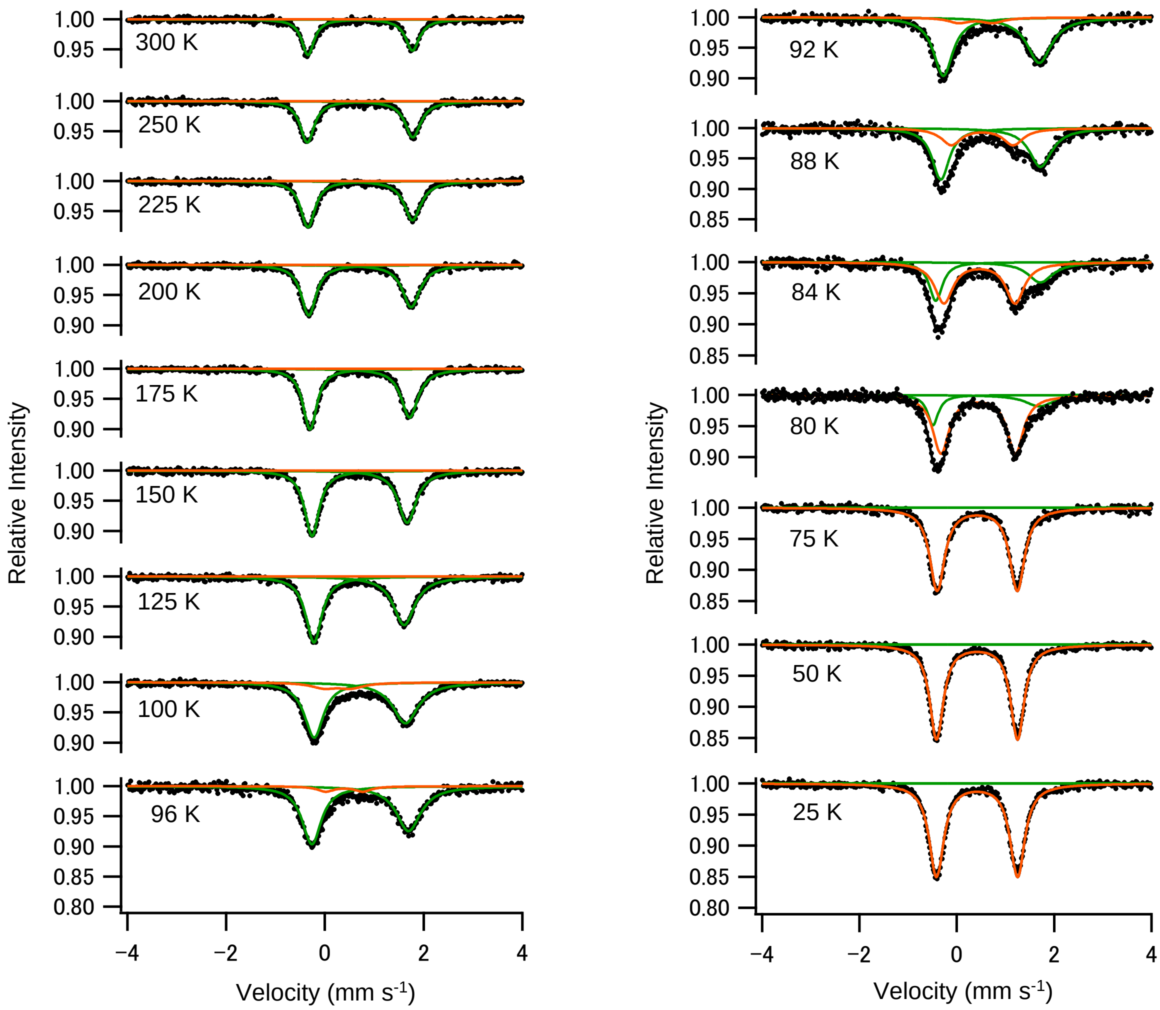

Supplementary Fig. 12 | Temperature dependent Mössbauer spectra of **1(PF$_6$)$_3$** between the temperature range of 300 to 25 K. Spin transition is observed at approximately 90 K. Upon heating above spin transition temperature, the QS value is slightly getting larger, which suggests that the contribution of $^{C}[Fe^{2+}_{HS}$–dhbq^{2}–$Co^{3+}_{LS}]$ state increases with increasing temperature at the high temperature phase.

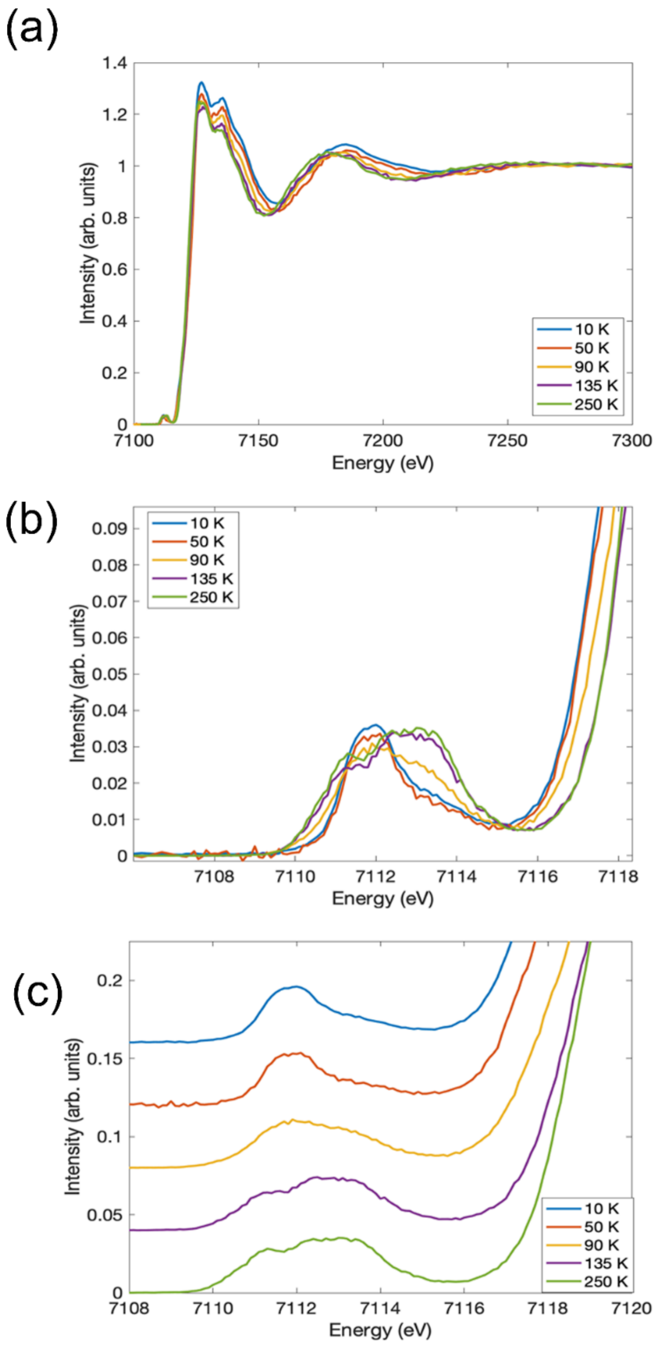

Supplementary Fig. 13 | (a) Variable-temperature HERFD-XANES measurement of $1(PF_6)_3$. (b) HERFD-XANES pre-edge structure measurement of $1(PF_6)_3$. (c) stacked diagram of HERFD-XANES pre-edge structure derived from (b).

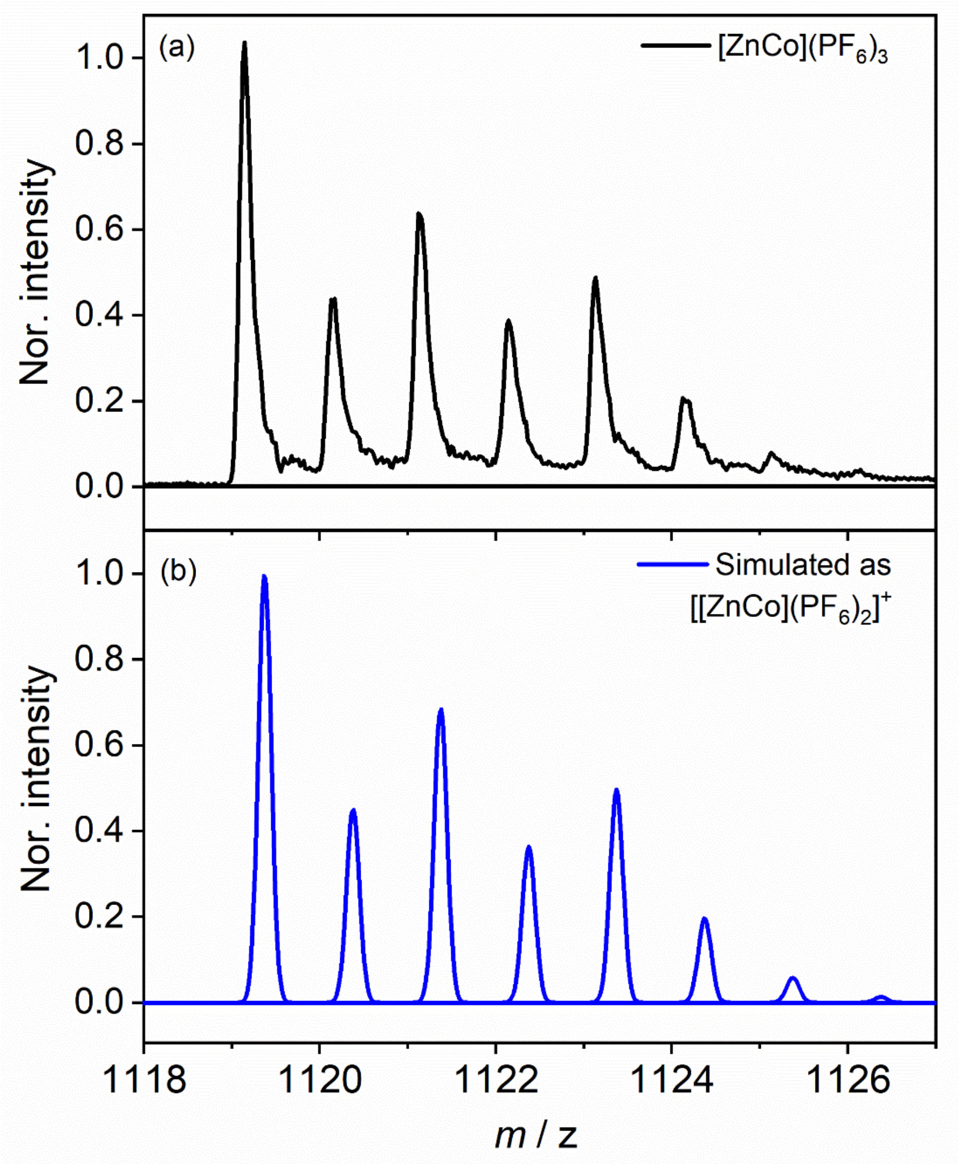

Supplementary Fig. 14 | ESI-MS spectrum of $[ZnCo](PF_6)_3$ in acetone. (a) The spectra exhibit the molecular peak of the $[\mathbf{ZnCo}](\mathbf{PF_6})_3$ (*m*/*z* = 1121.3). (b) Isotopic distribution simulation of $[[ZnCo](PF_6)_2]^+$($C_{38}H_{74}N_8O_4ZnCoP_2F_{12}$).

Supplementary Table 1 | Elemental analysis for 1(PF_6)$_3$

Metal elemental analysis:

	**Co**	**Fe**
**1st analysis**	4.72	4.49
**2nd analysis**	4.71	4.47
**Average value**	4.72	4.48

The composition ratio:

	**Co**	**Fe**
**1st analysis**	0.998	1
**2nd analysis**	0.999	1
**Average value**	0.998	1

Supplementary Table 2 | Crystallographic Data Collection and Structural Refinement Information for $1(PF_6)_3$

	$1(PF_6)_3$					
Empirical Formula	$C_{38}H_{74}CoF_{18}FeN_8O_4P_3$					
Formula Weight	1256.74					
Temperature(K)	60	100	150	200	250	300
Crystal System	*monoclinic*	*monoclinic*	*monoclinic*	*monoclinic*	*monoclinic*	*monoclinic*
Lattice Type	Primitive	Primitive	Primitive	Primitive	Primitive	Primitive
Space group	$P2_1$(#4)	$P2_1$(#4)	$P2_1$(#4)	$P2_1$(#4)	$P2_1$(#4)	$P2_1$(#4)
a(Å)	8.8140(7)	8.8712(3)	8.8898(3)	8.9174(3)	8.9381(3	8.9724(4)
b(Å)	29.745(2)	30.2203(9)	30.2852(9)	30.3617(8)	30.4465(8	30.5418(9)
c(Å)	10.1654(8)	10.1450(3)	10.1741(3)	10.2057(3)	10.2227(4	10.2409(4)
α(deg.)	90	90	90	90	90	90
β(deg.)	110.057(8)	110.049(4)	110.058(4)	110.037(4)	110.053(4	110.044(4)
γ(deg.)	90	90	90	90	90	90
V (Å^3)	2503.5(4)	2554.96(15)	2573.02(15)	2595.92(15)	2613.29(16	2636.36(19)
Z value	2	2	2	2	2	2
D_{calc}(g/cm^3)	1.667	1.634	1.622	1.608	1.597	1.583
radiation	MoKα (λ = 0.71073)					
R_1 (I>2.00s(I))	0.0485	0.0525	0.0523	0.0508	0.0508	0.0544
wR_2 (*all*)	0.1399	0.1301	0.1302	0.1308	0.1308	0.153
GOF	1.094	1.047	1.046	1.058	1.057	1.056
Flack parameter	0.15(2)	0.032(7)	0.039(9)	0.032(8)	0.034(8)	0.037(8)
CCDC	2025869	2025870	2025871	2025872	2025873	2025874

$^{a}R_1 = \Sigma||Fo| - |Fc||/\Sigma|Fo$. $^{b}R_2 = [\Sigma\{w(Fo^2 - Fc^2)^2\}/\Sigma\{w(Fo^2)^2\}]^{1/2}$

Supplementary Table 3 | Selected Bond Lengths (Å) for 1(PF_6)$_3$

	1(PF_6)$_3$					
	60 K	100 K	150 K	200 K	250 K	300 K
Fe1-O1 (Å)	1.904(5)	1.987(4)	2.009(4)	2.024(3)	2.035(4)	2.059(4)
Fe1-O2 (Å)	1.904(5)	1.991(5)	2.006(4)	2.021(4)	2.042(4)	2.048(4)
Fe1-N1 (Å)	2.038(5)	2.109(5)	2.124(5)	2.130(4)	2.135(5)	2.129(4)
Fe1-N2 (Å)	2.060(5)	2.145(5)	2.155(5)	2.162(4)	2.168(5)	2.164(5)
Fe1-N3 (Å)	2.026(5)	2.122(5)	2.139(5)	2.140(5)	2.133(4)	2.143(5)
Fe1-N4 (Å)	2.045(5)	2.143(5)	2.156(5)	2.165(5)	2.165(5)	2.173(5)
Co1-O3 (Å)	1.893(4)	1.893(4)	1.895(4)	1.901(4)	1.891(4)	1.905(4)
Co1-O4 (Å)	1.891(5)	1.891(5)	1.889(4)	1.893(4)	1.904(4)	1.891(4)
Co1-N5 (Å)	1.987(5)	1.979(5)	1.980(5)	1.983(4)	1.996(5)	1.988(5)
Co1-N6 (Å)	2.015(5)	2.020(5)	2.023(5)	2.024(5)	2.030(5)	2.023(5)
Co1-N7 (Å)	2.012(5)	1.996(5)	1.991(5)	1.993(5)	1.985(4)	1.990(5)
Co1-N8 (Å)	2.026(5)	2.024(5)	2.025(5)	2.029(5)	2.022(5)	2.032(5)

Supplementary Table 4 | ^{57}Fe Mössbauer spectral parameters for 1(PF_6)$_3$. IS (isomer shift), QS (quadrupole splitting) and LW (line width).

	Asymmetric doublet (high-temperature phase)				Symmetric doublet (low-temp. phase)			
T (K)	Fraction (%)	IS (mm s^{-1})	QS (mm s^{-1})	LW (left/right) (mm s^{-1})	Fraction (%)	IS (mm s^{-1})	QS (mm s^{-1})	LW (mm s^{-1})
300	100	0.715(2)	2.117(2)	0.298(8) /0.339(9)	0			
250	100	0.712(2)	2.139(2)	0.340(7) /0.387(8)	0			
225	100	0.715(2)	2.121(2)	0.348(6) /0.412(7)	0			
200	100	0.710(2)	2.077(2)	0.363(6) /0.431(7)	0			
175	100	0.707(2)	2.019(2)	0.335(4) /0.418(5)	0			
150	100	0.702(2)	1.928(2)	0.350(4) /0.441(6)	0			
125	100	0.690(2)	1.828(2)	0.390(4) /0.529(6)	0			
100	88(2)	0.705(2)	1.851(2)	0.466(8) /0.628(11)	12(2)	0.26(3)	0.55(5)	0.65(10)
96	93(2)	0.714(3)	1.960(3)	0.465(10)/0.60(2)	7(2)	0.39(3)	0.75(6)	0.42(9)
92	91(2)	0.708(3)	1.973(3)	0.477(10)/0.61(2)	9(2)	0.39(4)	0.69(7)	0.54(11)
88	72(3)	0.695(5)	2.050(5)	0.42(2) /0.56(2)	28(3)	0.521(13)	1.27(3)	0.50(4)
84	41(3)	0.642(10)	2.148(10)	0.33(2) /0.62(4)	59(3)	0.464(5)	1.463(13)	0.45(2)
80	26(2)	0.61(2)	2.18(2)	0.27(2) /0.71(7)	74(2)	0.438(3)	1.530(8)	0.403(10)
75	0				100	0.418(13)	1.644(3)	0.365(4)
50	0				100	0.4145(8)	1.662(2)	0.335(2)
25	0				100	0.4140(9)	1.664(2)	0.362(3)

Supplementary Table 5 | Crystallographic Data Collection and Structural Refinement Information for [ZnCo](PF_6)$_3$

	**[ZnCo](PF_6)$_3$**	
Empirical Formula	$C_{38}H_{74}CoF_{18}ZnN_8O_4P_3$	
Formula Weight	1266.26	
Temperature(K)	100	250
Crystal System	*monoclinic*	*monoclinic*
Lattice Type	Primitive	Primitive
Space group	$P2_1$(#4)	$P2_1$(#4)
a(Å)	8.8920(2)	8.9675(3)
b(Å)	30.4196(5)	30.5614(6)
c(Å)	10.2096(3)	10.1886(3)
α(deg.)	90	90
β(deg.)	110.326(3)	110.067(4)
γ(deg.)	90	90
V (Å^3)	2589.64(12)	2622.77(14)
Z value	2	2
D_{calc}(g/cm^3)	1.624	1.603
radiation	MoKα (λ = 0.71073)	
R_1 (I>2.00s(I))	0.0389	0.0368
wR_2 (*all*)	0.0998	0.0968
GOF	1.027	1.057
Flack parameter	0.010(4)	0.001(4)
CCDC	2025875	2025876

$^aR_1 = \Sigma||Fo| - |Fc||/\Sigma|Fo$. $^bR_2 = [\Sigma\{w(Fo^2 - Fc^2)^2\}/\Sigma\{w(Fo^2)^2\}]^{1/2}$

References:

1 Sheldrick, G. M. Crystal structure refinement with SHELXL. *Acta Crystallographica Section C Structural Chemistry* **71**, 3-8 (2015).

2 Dolomanov, O. V. OLEX2: a complete structure solution, refinementand analysis program. *J. Appl. Cryst.* **42**, 339-341 (2009).

3 Gaussian 09, Revision E.01, Frisch, M. J.; Trucks, G. W.; Schlegel, H. B.; Scuseria, G. E.; Robb, M. A.; Cheeseman, J. R.; Scalmani, G.; Barone, V.; Mennucci, B.; Petersson, G. A.; Nakatsuji, H.; Caricato, M.; Li, X.; Hratchian, H. P.; Izmaylov, A. F.; Bloino, J.; Zheng, G.; Sonnenberg, J. L.; Hada, M.; Ehara, M.; Toyota, K.; Fukuda, R.; Hasegawa, J.; Ishida, M.; Nakajima, T.; Honda, Y.; Kitao, O.; Nakai, H.; Vreven, T.; Montgomery, J. A., Jr.; Peralta, J. E.; Ogliaro, F.; Bearpark, M.; Heyd, J. J.; Brothers, E.; Kudin, K. N.; Staroverov, V. N.; Kobayashi, R.; Normand, J.; Raghavachari, K.; Rendell, A.; Burant, J. C.; Iyengar, S. S.; Tomasi, J.; Cossi, M.; Rega, N.; Millam, J. M.; Klene, M.; Knox, J. E.; Cross, J. B.; Bakken, V.; Adamo, C.; Jaramillo, J.; Gomperts, R.; Stratmann, R. E.; Yazyev, O.; Austin, A. J.; Cammi, R.; Pomelli, C.; Ochterski, J. W.; Martin, R. L.; Morokuma, K.; Zakrzewski, V. G.; Voth, G. A.; Salvador, P.; Dannenberg, J. J.; Dapprich, S.; Daniels, A. D.; Farkas, Ö.; Foresman, J. B.; Ortiz, J. V.; Cioslowski, J.; Fox, D. J. Gaussian, Inc., Wallingford CT, 2009.

4 Wachters, A. J. H. Gaussian Basis Set for Molecular Wavefunctions Containing Third-Row Atoms. *J. Chem. Phys.* **52**, 1033-1036 (1970).

5 Hay, P. J. Gaussian basis sets for molecular calculations. The representation of 3d orbitals in transition-metal atoms. *J. Chem. Phys.* **66**, 4377-4384 (1977).

6 Dunning, T. H. Jr. & Hay, P. J. in Modern Theoretical Chemistry, Ed. Schaefer, H. F. III, Vol. 3 (Plenum, New York, 1977) 1-28.

7 Becke, A. D. Density-functional thermochemistry. III. The role of exact exchange. *J. Chem. Phys.* **98**, 5648-5652 (1993).

8 Reiher, M. Theoretical Study of the $Fe(phen)_2(NCS)_2$ Spin-Crossover Complex with Reparametrized Density Functionals. *Inorg. Chem.* **41**, 6928-6935 (2002).

9 Yamamoto, K.; Kawasaki, A.; Chinen, T.; Ryugo, A.K. Temperature-Modulated Pyroelectricity Measurements of a Thin Ferroelectric Crystal With in-Plane

Polarization and the Thermal Analysis Based on One-Dimensional Layer Models. Preprints 2021, 2021060190 (doi: 10.20944/preprints202106.0190.v1).
